# Impaired Melatonin Secretion, Oxidative Stress and Metabolic Syndrome in Night Shift Work

**DOI:** 10.3390/antiox12040959

**Published:** 2023-04-19

**Authors:** Sorina Hohor, Cristina Mandanach, Andreea Maftei, Corina Aurelia Zugravu, Marina Ruxandra Oțelea

**Affiliations:** 1 Doctoral School, “Carol Davila” University of Medicine and Pharmacy, 37 Dionisie Lupu Street, Sector 2, 020021 Bucharest, Romania; sorina.giurgiulescu@drd.umfcd.ro (S.H.); cristina.paraschiv@drd.umfcd.ro (C.M.); andreea.mutu@drd.umfcd.ro (A.M.); 2 “Dr. Carol Davila” Central Military Emergency University Hospital, 134 Calea Plevnei, Sector 1, 010242 Bucharest, Romania; 3 Department of Hygiene and Ecology, “Carol Davila” University of Medicine and Pharmacy, 37 Dionisie Lupu Street, Sector 2, 020021 Bucharest, Romania; corina.zugravu@umfcd.ro; 4 Clinical Department 5, “Carol Davila” University of Medicine and Pharmacy, 37 Dionisie Lupu Street, Sector 2, 020021 Bucharest, Romania

**Keywords:** metabolic syndrome, night shift, melatonin, oxidative stress, chronodisruption

## Abstract

Metabolic syndrome has been associated in many studies with working in shifts. Even if the mechanistic details are not fully understood, forced sleep deprivation and exposure to light, as happens during night shifts, or irregular schedules with late or very early onset of the working program, lead to a sleep–wake rhythm misalignment, metabolic dysregulation and oxidative stress. The cyclic melatonin secretion is regulated by the hypothalamic suprachiasmatic nuclei and light exposure. At a central level, melatonin promotes sleep and inhibits wake-signals. Beside this role, melatonin acts as an antioxidant and influences the functionality of the cardiovascular system and of different metabolic processes. This review presents data about the influence of night shifts on melatonin secretion and oxidative stress. Assembling data from epidemiological, experimental and clinical studies contributes to a better understanding of the pathological links between chronodisruption and the metabolic syndrome related to working in shifts.

## 1. Epidemiological Data

Working evenings, nights, or on weekends can have a negative impact on a worker’s physical health and well-being [1]. Shift workers and former shift workers with more than 10 years occupational history of rotating shifts are more likely to have metabolic syndrome (MetS) than workers who never worked shifts [2,3,4]. In a recent meta-analysis, the risk for MetS was found to be increased by 12%, with slightly more prevalence in women [5].

Other studies have showed an increased incidence in one or several components of MetS such as a high waist circumference, elevated blood pressure, blood triglycerides and glucose levels, or low HDL cholesterol in shift workers compared with day workers [6,7,8]. Even more so, night shift work was associated with a high normal weight obesity, defined as a larger than normal percentage of fat mass, despite a normal BMI [9]. There is a wide variation of these components according to regions, race, age and gender, making comparisons more difficult and requiring a more nuanced approach of these data [10,11,12,13].

Gender differences have benefited from much attention, as MetS has anyway a gender-specific criteria; however, a recent meta-analysis did not find any difference in obesity according to gender [14]. A long-term cohort study suggested a positive association between rotating night shift work and type 2 diabetes risk, irrespective of the age group [15]. In conglomerated data from two long-term cohorts, a multivariable adjusted hazard ratio for type 2 diabetes was 1.31 (95% CI 1.19–1.44) per five-year increment of duration of rotating night shift work. This risk increased with the joint association of night shift with unhealthy lifestyle factors to 2.83 (95% CI 2.15–3.73) [16]. In another study, an irregular daily routine was associated with increased risks of total cardiovascular disease (HR 1.25, 95% CI 1.10–1.41), total stroke (HR 1.19, 95% CI 1.04–1.36), and coronary heart disease (HR 1.60, 95% CI 1.17–2.20) in women [17].

A summary of the relevant epidemiological studies is presented in Supplementary Table S1.

In MetS, oxidative stress was proposed as a unifying mechanism of metabolic and cardiovascular disorders [18]; therefore, in this review, we will present the links between a disrupted circadian rhythm and an abnormal oxidative status. As melatonin (MT) is a central regulator of the circadian oscillations and has a well-defined antioxidant role, the second part of this the presentation will focus on the connection between the impairment of MT secretion and oxidative stress.

## 2. Oxidative Stress and Working in Shifts

Despite this sound epidemiological evidence, the specific mechanism of the interaction between working in shifts and MetS is not fully understood. The fact that metabolism has circadian oscillations has been documented for a long time now, both in animal and human studies. The central clock, located in the suprachiasmatic nucleus, presents intrinsic oscillations of the Brain- and muscle ANRT-like protein-1 (BMAL1) and Circadian Locomotor Output Cycles Kaput (CLOCK) that regulates the central expression of many proteins involved in metabolism regulation, such as glucocorticoid, insulin, and various adipokines [19,20]. A sophisticated central feedback control mechanism, involving those such as the period circadian regulator 2 (PER2) and cryptochromes (CRYs) complex and REV-ERBs products is partially self-regulated but also dependent on other external influencers (e.g., light, melatonin, exercise, nutrition, etc.) among which light plays a central role [21]. Within a certain individual dependent variability, sleep deprivation, which happens in night shift, reduces the light-induced electrical activity within the suprachiasmatic nuclei in humans [22]. The effect of light is not limited to the central clock. In a night shift simulated experiment, PER1 and BMAL1 rhythms were delayed in peripheral mononuclear blood cells [23]. A later light exposure and timing of meals, events which are present in night shifts, were associated with a shift of 0.60 to 1.39 h to later bedtimes [24]. Exercise influence, comprehensively revised elsewhere [25], has beneficial influences in the stabilization of the circadian rhythm. Nutrition and exercise should be considered not only for their influence of the circadian genes, but also for their potential to generate oxidative stress. For example, short duration, moderate exercise does not increase oxidative stress, while high-effort and endurance training results in a higher ROS production, which is balanced by an increase in antioxidant enzymes. Most studies explain the beneficial effect of exercise on multiple chronic diseases related to oxidative stress by the hormesis pattern of the effect of exercise on human physiology in which a transient increase in low levels of a stressor is beneficial for the functionality of the cells, whereas a high dose damages the cellular mechanisms [26].

The central clock also provides input for the peripheral expression of the clock genes. The peripheral circadian clock genes exist in almost all cells of the body and have influences on the cellular metabolism. CLOCK-BMAL1 affects the transcription of genes involved in fatty acids and cholesterol synthesis, while PER2-CRY influences the transcription of genes participating to the cell stress reaction, carbohydrate and lipid metabolism and to the cell cycle, including adipogenesis [19,27].

**Figure 1:**
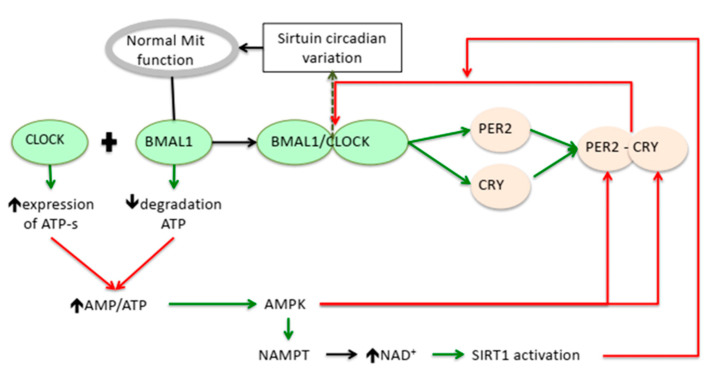
Putative relation between circadian genes regulation and modulators of the oxidative stress. On one side, BMAL1 and CLOCK regulate the mitochondrial function and increases the AMP/ATP ratio. On the other side, the circadian expression of *BMAL1* and *CLOCK* genes is regulated by adenosine monophosphate activated protein kinase acting on the PER-2-CRY expression and indirectly by activating sirtuin 1. Legend: BMAL-1: Brain- and muscle ANRT-like protein-1; CLOCK: Circadian Locomotor Output Cycles Kaput; PER2: period circadian regulator 2; CRY: cryptochromes; Mit: mitochondria; ATP-s: ATP-synthase; AMP: Adenosine monophosphate; ATP: Adenosine triphosphate; AMP-kinase: adenosine monophosphate activated protein kinase; NAD: Nicotinamide adenine dinucleotide; NAMPT: nicotinamide phosphoryl-transferase; SIRT1: Silent Information Regulator 1. 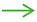 Activation; 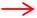 Suppression.

Beside the transcriptional interference at a nuclear level, the clock genes influence the **NAD(P)/NAPH redox system** which participates in the control of the oxidative status of the cells. The reduced form of nicotinamide adenine dinucleotide (NADH) is an important donor of electrons for ROS formation in mitochondria and for NADH oxidases in cytosol, but it also supplies H^+^ for the activity of the antioxidant enzymes, namely, for the glutathione reductases (GRs) and thioredoxin reductases (TrxRs) [28]. A loss of BMAL1 activity in the liver was proven to cause the swelling of mitochondria. Meanwhile, impairment of the diurnal rhythms of the Silent Information Regulator 3 (SIRT3), affects the mitochondrial oxidative phosphorylation [29] and increases the production of ROS (Figure 1).

The influence is reciprocal, as NAD^+^ dependent SIRT1 creates a negative feedback loop to the central clock genes [30]. In aged wild mice, SIRT1 levels in the suprachiasmatic nucleus decreases in parallel with those of BMAL1 and PER2 [31]. In hepatic and fibroblast cells, SIRT1 binds to CLOCK-BMAL1 heterodimers and promotes the deacetylation and degradation of PER2, blocking the negative loop initiated by PER2 on the BMAL-1 transcription [30] (Figure 1). Together with the regulatory function of SIRT1 on the transcription of various proteins involved in the control of the metabolism, the NAD^+^/SIRT/Clock genes contribute to the nutrient handling and the redox status.

The **adenosine monophosphate activated protein kinase** (AMPK) is the central regulator of the cellular energy balance. AMPK is activated by an increase in the AMP/ATP ratio, adiponectin, exercise, ghrelin and some pharmacological agents [32]. AMPK promotes catabolic pathways to generate more ATP, and inhibits the anabolic ones. Circadian genes are substrates for phosphorylation by AMPK as part of the integration between feeding, metabolic homeostasis and the circadian rhythm [33]. CLOCK and BMAL1 transcriptional effects increase ATP levels directly via the ATP-synthetase expression [34] or, indirectly, by reducing the uncoupling protein 2 in certain tissues, including in the pancreatic islet cells [35]. The balance between ATP/ADP is dependent on the nutrient substrate availability and is reflected by the level of the AMP-activated protein kinase (AMPK), which is also a regulator of the CRY and PER proteins levels [33,36]. All these are relevant for the metabolic side of MetS.

For the cardiovascular component of MetS, it has to be underlined that AMPK has a significant role in the physiology of the endothelia [37]. In the absence of AMPK, nitric oxide (NO) production by endothelial nitric oxide synthase (eNOS) and the nuclear factor erythroid 2–related factor 2 (Nrf2) expression are reduced [38]. There is also a failure to improve the antioxidant capacity induced by exercise [38], an attenuation in the expression of superoxide dismutase [39] and a higher oxidant activity induced by angiotensin II [40]. In the presence of peroxinytrite, an overexpression of AMPK contributes to the formation of O_2_^−^ from eNOS [41], which makes an argument on the conditional antioxidative effect of AMPK.

**Mitochondrial dynamics** reflect changes in the morphology of mitochondria (e.g., fusion or fragmentation) according to the metabolic status of the cells. They are tissue specific and regulated by the availability of a substrate, a constellation of hormones and adrenergic stimulation. The fusion state generally corresponds to better functionality, with better efficiency in coupling the substrate oxidation with energy production [42]. Mitochondrial dynamics have circadian rhythmicity, which has been observed in cultured cells and seem to be regulated by the circadian genes, such as PER1/2, D-Box Binding PAR BZIP transcription factor (DBP1) [43] or BMAL [36] genes. A disruption of the normal clock machinery affects the energy substrate handling in the cells and the production of ROS. At a central level, in the proopiomelanocortin (POMC) neurons, an altered mitochondrial dynamic was normalized by antioxidants. In this experiment, the reduction in glucose-stimulated insulin secretion was directly linked to the ROS production in POMC neurons [44].

Results from **clinical studies** confirm the hypothesis that a night shift has pro-oxidant effects. At the end of the nightshift, there was an increase in the total oxidant status and a decrease in the antioxidant status [45]. In this study, the total antioxidant status was measured by spectrophotometry. This method is based on the reaction of the oxidants present in the sample with a ferrous ion-o-dianisidine complex. There was also another biomarker of oxidative stress which was increased at the end of the night shift: the urinary 8-oxo-7,8-dihydro 2′deoxyguanosine (8-oxoGua) [46]. The 8-oxoGua is a product of the oxidative damage to 2′-deoxyguanosine, which is removed from the DNA by 8-oxoguanine DNA glycosylase 1 (8-oxoGDG); an increase in the urinary concentration of 8-oxoGua reflects either an increase in oxidative stress or in the DNA repair, or both. This might explain why in another study, the 8-oxoGua was found to be decreased when measured immediately after a night shift and increased in the second day after the night shift [47], probably reflecting the delay in the onset of the reparatory mechanisms. In this second study, MT had a similar variation with 8-oxoGua. In a study which included 397 employees from nine police stations, oxi-LDL, neutrophil gelatinase lipocalin-2 (NGAL-2), ferritin, protein C reactive and HOMA-IR were increased during a night shift [48]. The common finding in these studies is that oxidative stress is present in shift work, independent of a pathological status.

There are few studies focused on the oxidative status in MetS related to chronodisruption. In a medium size cross-sectional study, significantly lower values of antioxidants were found in night workers without clinical criteria of MetS compared to day workers. The antioxidants measured in this investigation were ferric reducing/antioxidant power, catalase (CAT) and superoxide dismutase (SOD) [49]. Malondialdehyde (MDA) was measured in healthy, middle-aged individuals during the day; the conclusion of this study was that the endogenous circadian system increases MDA in the vulnerable morning hours for cardiovascular events [50].

## 3. Melatonin Dysregulation—A Link between Oxidative Stress, Metabolic Syndrome and Working in Shifts

Melatonin (MT) is considered a “biological time–domain molecule”, with a circadian, seasonal and even transgenerational variation [51]. A meta-analysis showed reduced MT levels in night shift workers, particularly in those with a fixed schedule [52], explaining, at least in part, the disorders associated with the circadian rhythm disruption.

The chronodisruption induced by night shifts was the basis for developing studies about the relation between MetS and MT modifications. In women with MetS, the level of night MT was significantly lower and the circadian variation was blunted [53]. This low amplitude of variation was inversely correlated with all the components of MetS. In another research study, the night MT/insulin ratio was positively correlated with HDL-cholesterol and negatively with the LDL-cholesterol and total cholesterol. In the MetS patients included in this study, but not in the controls, the lower difference between the night and day MT was associated with higher daily insulin and with higher glucose [54]. Similar blunted MT values were found in the non-dipping hypertensive patients [55]. A long-term study of a cohort of 554 women showed that the first morning urinary levels of 6-sulphatoxy-melatonin aMT6, an accurate measure of cumulative nocturnal plasma melatonin levels, in the recruitment phase, were independently and inversely associated with incident hypertension [56]. This is an argument that low endogenous MT might be a predictor of the future development of hypertension.

A placebo-controlled singe-blind study in healthy adults [57] described significant differences in the effects of 5 mg of MT administered in the morning and in the evening. In both situations, the tolerance to glucose was impaired, but the impairment was more pronounced when administered in the morning. In the morning, the MT decreased insulin sensitivity, while in the evening the MT decreased insulin release. These data indirectly support the hypothesis that chronodisruption and the phase advance or phase delay in MT secretion might have significant metabolic consequences. This metabolic effect might be related with the phase advance circadian rhythms after exogenous MT administration in the evening and a phase delay if administrated in the morning [58].

Overall, these results indicate that MT is associated with MetS-related disorders. The shifts from the normal circadian rhythm of the secretion of MT interfere with metabolism and cardio-vascular functions. This chronodisruption is congruent with the conclusion of a systematic review that looked for the effects of light exposure at night and MT secretion [59]. The authors concluded that both a suppression of MT and a reduction in the overall MT amplitude of variation are present. Interestingly, these effects were recorded in night shift workers even for an exposure below 80 lx, which is considered the threshold for the activation of the circadian system, and the MT reduction was cumulative with the number of successive nights worked. An experimental study showed that after exposure to 4 h of bright light before sleep, as might happen in night work, the total antioxidant activity was notably increased [60]

### 3.1. Melatonin—An Antioxidant Molecule

Melatonin is an indoleamine derivate of tryptophan. The amino acid is converted in serotonin, then acetylated to N-acetylserotonin (NAS) by serotonin N-acetyltransferase and finally converted into melatonin by N-acetylserotonin-O-methyltransferase.

**Figure 2:**
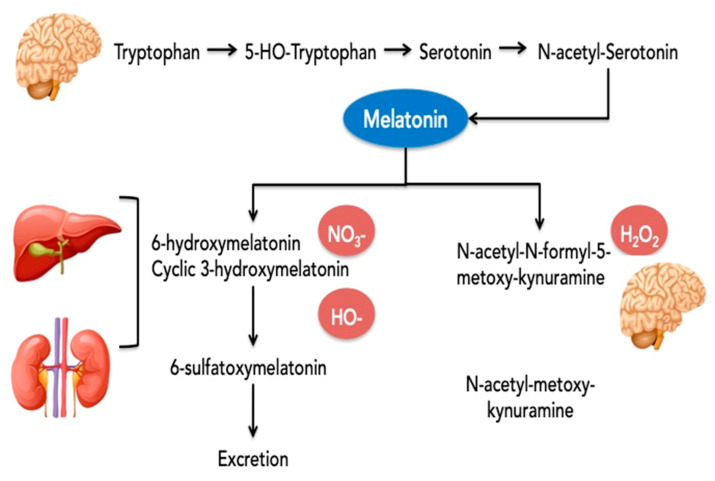
Metabolism of melatonin and the predominant targets of the different metabolites for the antioxidative effect.

The production of melatonin follows a rhythmic pattern with secretory peaks and distribution in night time. These peaks are reflected in various body tissues and organs [61]. The synthesis of MT was detected in the pineal gland and in multiple extrapineal tissues including the brain, retina, lens, cochlea, Harderian gland, airway epithelium, skin, gastrointestinal tract, liver, kidney, thyroid, pancreas, thymus, spleen, immune system cells, carotid body, reproductive tract, and endothelial cells. The levels of MT in extrapineal tissues and/or body fluids could be even higher than in the pineal gland or blood levels. For example, the cerebrospinal fluid levels are 20-fold higher than plasma concentrations [62]. In gastrointestinal tissues the levels are higher by 400 times compared to the pineal gland and are probably related to the periodicity of food intake [63]. The half-life of melatonin in circulation is 20–50 min. After synthesis, MT persists in the human body for 4 to 5 h [64]. Approximately 1% of blood melatonin is excreted in urine without being metabolized [65]. In brief, the metabolism of MT and the main targets as the antioxidants of these metabolites are presented in Figure 2.

Legend: the main metabolites resulting from single-electron transfer reactions by MT are: (1) cyclic 3-hydroxymelatonin (c3OHM) from scavenging two hydroxyl radicals; (2) N-acetyl-N-formyl-5-methoxykynuramine (AFMK) from detoxifying hydrogen peroxide or a termination product of radical chain reactions where it is metabolized to N-acetyl-methoxykynuramine and both are present predominantly in the brain; and (3) 6-hydroxymelatonin from the reaction of melatonin with activated peroxynitrous acid [66]. The 6-sulfatoxymelatonin (6-SMT) is the main final urinary metabolite, but Cyclic 3-hydroxymelatonin (c3OHM) was also detected in human urine, especially when melatonin was given exogenously or after an imposed oxidative stress (e.g., exposure to ionizing radiation). Because of that, c3OHM is considered a general marker of oxidative stress [67]. Cyclic 3OHM is also a potent free radical scavenger that can be converted by two hydroxyl radicals to another key metabolite of melatonin, namely, *N*^1^-acetyl-*N*^2^-formyl-5-methoxykynuramine (AFMK). Some of the CYTPs—CYP2C19 and to a much smaller extent, CYP1A2, demethylate melatonin to its precursor, N-acetylserotonin (NAS).

The nitrosation of MT leads to the formation of *N*-nitrosomelatonin. This reaction was observed with various NO donors and also with peroxynitrite in liver, kidney and brain tissue. The advantage of *N*-nitrosomelatonin is its ability to release both melatonin and NO*, an easily diffusible free radical [68].

AFMK and AMK scavenge reactive oxygen and nitrogen species [69], but AFMK is generally less reactive than MT and AMK. AMK interacts more rapidly, indicating an even higher reactivity towards the carbonate radical than melatonin and it is also a better singlet oxygen scavenger than melatonin. It is very interesting that in an interaction with reactive nitrogen species, AMK generates a stable compound, 3-acetamidomethyl-6-methoxy-cinnolinone (AMMC), which does not spontaneously re-donate NO and, thus, becomes a more effective inhibitor of NO formation than melatonin [70].

In conclusion, all three metabolites of melatonin (i.e., c3OHM, AFMK, and AMK) have antioxidant effects, prolonging the direct beneficial effect of melatonin [66].

**Figure 3:**
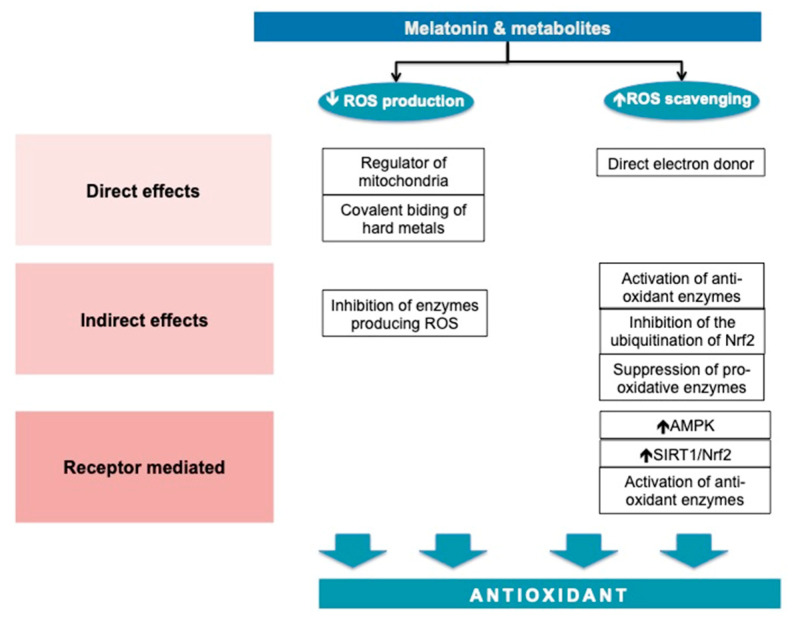
Melatonin—an antioxidant molecule.

MT has direct, indirect and receptor-mediated effects. MT acts by reducing the formation of ROS and in increasing the defense capacity against them. The antioxidant effects are summarized in Figure 3.

MT is highly prevalent [71,72] and has a significant role in mitochondrial homeostasis. MT maintains the electron flow, increases the activity of the respiratory chain complexes I and IV [73] and decreases electron leakage [74]. MT interferes with mitochondria fusion in circumstances associated with oxidative stress, such as ischemia-reperfusion of the myocardium by the mRNA and the protein expression of the transcription of optic atrophy 1. In this experiment, the mitochondrial fusion was responsible for the reduction in ROS in cardiomyocytes [75].

MT and its metabolites are direct free radical scavengers to most of the free radicals produced inside the cells, with certain specific affinities for substrates. MT does not appear to be a potent direct scavenger of the superoxide anion radical, but is more effective as a scavenger of hydroxyl radicals than its metabolites (5-methoxytryptamine) or precursors (N-acetylserotonin and serotonin). MT reduces lipid peroxidation during oxidative challenges probably by delaying the initiation of lipid peroxidation, rather than direct scavenging of the peroxyl radical [76]. In combination with metal-chelating, the scavenging antioxidative effect is augmented [77]. 

MT acts through two types of receptors, namely, MT1 and MT2. The MT1 receptor pathway is better characterized. The MT binding to MT1 initiates a cascade of cellular events, which lead to the translocation of SIRT3 in the mitochondria-associated membranes, the zone in which mitochondria interacts with the endoplasmic reticulum [78]. SIRT3 interacts with at least 84 mitochondria proteins through which it contributes to the homeostasis of mitochondria and metabolic stability [79]. MT1 signaling through SIRT1 activates Nrf2, increasing the transcription of antioxidant enzymes [80].

Beside these direct effects, MT acts indirectly to restore the oxidative equilibrium by the stimulation of antioxidant enzymes including SODs, glutathione peroxidase (GSH-PX), glutathione reductase (GSR) and catalase, and the suppression of pro-oxidant enzymes such as nitric oxide synthases (NOS), lipoxygenases, and presumably also quinone reductase 2 [74]. Basically, MT increases the mRNA levels for manganese SOD, copper–zinc SOD, and glutathione peroxidase [81,82], and it enhances intracellular glutathione (another important antioxidant) levels. The last effect is obtained via the stimulation of gamma-glutamylcysteine synthase, the rate-limiting enzyme in the synthesis of glutathione [83]. MT also regulates Nrf2, which binds in the nucleus to ARE (the antioxidant response element) on the promotor region of SOD, GSH-PX [84].

In what concerns MetS and its related diseases, in experimentally-induced type 2 diabetes mellitus, MT prevented the development of oxidative stress and sustained the levels of glutathione and glutathione peroxidase activity in the heart and pancreas of rats [85]. In another study, MT downregulated serum glucose levels and improved the levels of antioxidants such as glutathione, glutathione peroxidase and glutathione reductase in the brains of diabetic rats [86]. Urata Y et al. showed that this protective action of MT against oxidative stress is also present in vascular endothelial cells [87]. MT appears to be a versatile hepatoprotective in models of experimental liver injury in rats, mice and chicks. MT strongly attenuated hepatic lipid peroxidation (LPO), by reducing the levels of oxidized glutathione (GSSG) and enhancing the activity of the antioxidant enzyme glutathione peroxidase (GSH-PX) in both the brain and liver [88].

MT has multiple positive effects on the components of MetS. In the following paragraphs we will restrict the presentation only to the antioxidative processes.

### 3.2. Antioxidative Effects Mediated by MT in Obesity

Adipose tissue is one of the main sources of ROS in MetS. As it is well established, adipose tissue is not homogenous. Obesity represents the accumulation of white fat, and particularly for MetS, the accumulation is more abundant in the visceral area. Browning the white adipocytes is a novel approach to treat obesity [89] and MT has been shown to have this effect [90]. In human and rat lipoaspirates, the differentiation of mesenchymal cells in contact with MT was deviated towards an increased uncoupling protein 1 (UCP1), Cbp/P300 Interacting Transactivator with Glu/Asp Rich Carboxy-Terminal Domain 1 (CITED1), and Peroxisome proliferator-activated receptor-gamma coactivator (PGC)1-α expression, which characterize beige and brown adipocytes [91]. In another experiment as well, MT upregulated the expression of thermogenic genes: *Ucp1*, *Prdm16*, *Elovl3*, and *Cidea* [92]. Of note, UCP1 inhibits mitochondrial ROS production [93] probably by lowering the mitochondrial membrane potential [94].

In other experiments, MT induced a lower expression of NADPH oxidase subunits (p^22phox^, p^67phox^, Nox4) in epididymal white adipose tissue [84]. The epididymal fat is similar to the fat in the mesenteric, mediastinal, retroperitoneal and perirenal, forming all together the visceral adipose tissue of a rat; therefore, the action of MT on epididymal fat is relevant in the context of experimental MetS [95]. With MT administration, obese mice improved their redox balance, and they showed a reduction in MDA in the plasma, in the adipose tissue and liver and an increase in the plasma glutathione concentration [96].

MT has also increased the level of SOD and reduced the mitochondrial nitrate level, in Zücker diabetic fatty rats, beside an overall improvement in the mitochondrial activity related to the restoration of an efficient respiratory control [97]. Other antioxidant enzymes which were decreased by ahigh fat diet in mice (*SOD*, *Cat*, *GPX1* activity) were restored by a MT treatment [92].

Obese mice had a decrease in O_2_ consumption. After treatment with MT, this pathological mechanism was significantly reduced. The functional restoration was probably related to the scavenger function of MT, but also by the upregulation of Peroxisome Proliferator-Activated Receptor Gamma Coactivator 1-alpha (Pgc1alfa) and PR-domain containing 16 (Prdm16), which act as co-regulators of the expression of mitochondrial genes [98].

Overall, these studies show the role of MT in the restoration of the oxidative status in adipose tissue from obese animals. There are much fewer studies on the desynchronization of the circadian rhythm; however, a very comprehensive research study on the effects of forced internal desynchronization induced by a period of T-cycles of 22 h of male Wistar rats showed a significant decrease in HDL-cholesterol, a raise in LDL-cholesterol, a decrease in SOD and CAT protein expression and a reduction in baroreflex sensitivity [99]. This experiment indicates that cardio-metabolic impairment can be reproduced in rats and, indirectly confirms the experiments described above. Nevertheless, the extrapolation to human functionality remains, as always, an open question.

### 3.3. Antioxidative Effects Mediated by MT in the Vascular Wall

Maintenance of the endothelium and the aortic wall dimension are influenced by MT treatment [100]. Regarding these effects there is a general agreement, but deciphering the anti-oxidant contribution to vascular wall homeostasis is still controversial. For example, in aortic ring homogenates pre-incubated in noradrenaline, phenylephrine, acetylcholine and sodium nitroprusside, MT had an effective endothelial vasorelaxant effect, conjoined with a reduction in lipid peroxides and an elevation in NO, cGMP, glutathione, and of the SOD activity [101]. In another experiment, an MT receptor agonist (2-iodomelatonin) inhibited the α1-adrenergic-induced vasoconstriction but the vasoactive effect was not related to the antioxidants (i.e., SOD, tempol, and CAT) [102]. This latest experiment also showed that the removal of the endothelium enhanced the vasodilator effect of MT. This result overlaps to the anti-oxidative effect of MT, which scavenges not only excessive ROS but also various endothelium-derived relaxing factors, such as nitric oxide, and peroxynitrite [103].

Regarding NO, in vitro experiments showed that MT reduces the NO production [104]. In others, the eNOS activity and the NO levels were enhanced [105,106]. In this last experiment, MT also activated the large conductance Ca^2+^-activated K^+^ channels (BK_Ca_) channels, which hyperpolarize the cell and oppose the vasoconstriction. A reduction in COX-2 and inducible NO synthase (iNOS) activity were also reported in hypertensive rats treated with MT, leading to a significant improvement of the endothelial function, measured as a reduction in the wall tension with acetylcholine [107].

An interesting study for the circadian rhythm disruption was conducted in mice with hypertension due to an inhibitor of NO-synthetase (L-NAME). The MT daily rhythm was measured during the day and compared to normotensive rats. There was a much higher night-time peak of MT in the L-NAME group, which suggests that NO might have an inhibitory effect on the pituitary MT secretion [108].

The vascular suppleness, however, is not only the result of the vasoactive molecules or of the nervous input. It highly depends on the inflammatory process in the arterial wall which finally leads to atherosclerosis. Atherogenesis starts from the endothelial injury induced by oxidative stress and inflammation, in which oxidized low-density lipoprotein (ox-LDL) has a central role. It seems that MT could interfere with the prevention, the initiation, and the progression of atherosclerosis plaque. A 2019 experimental study on apolipoprotein E (ApoE)-deficient male mice divided them in four groups: the control group, cholesterol-fed group, prevention group (cholesterol diet/MT) and treatment group (cholesterol diet/MT). The results showed that the administration of MT (for both purposes, namely, prevention and treatment) dramatically reduced the atherosclerotic plaque area. Additionally, MT significantly reduced the ratio of collagen content to the total aortic lesion area compared with that in the cholesterol-fed group. In the treatment group, only the atherosclerotic plaque area was reduced, with no effect on the collagen content [109]. Cultured endothelial cells exposed to ox-LDL gradually lose their capacity to proliferate and to migrate. MT added to the cell culture reversed these dysfunctionalities and prevented the upregulation of caspase-3 activity and apoptosis. The ox-LDL-induced mitochondrial oxidative stress was also strongly inhibited by MT [110].

Another possible mechanism of the intervention of MT in the atherosclerotic process is related to the inhibition of the nuclear factor kappa light chain enhancer of activated B cells (NF-kB) pathway. MT increases the expression of the inhibitor of the NF-kB (IκB), maintaining NF-kB in the cytoplasm and therefore preventing the transcription of pro-inflammatory and pro-oxidative factors [111]. This inhibition also downregulates Runx2 transcription, a critical factor for the calcification of the vascular extracellular matrix [112]. The consequence of the NF-kB inhibition might also be the activation of autophagy [113,114].

This apoptotic effect was noticed in injured brain tissues [115,116]. It is also known that ROS induces the Receptor-Interacting Protein Kinase 3 (Ripk3) cascade, Drp1 activation, Bax-dependent cytochrome c [114] and apoptosis. MT is able to reduce ROS but also to block the Ripk3 and Drp1 pathways, preventing apoptosis. This has been proposed as one of the protectives mechanisms in ischemia-reperfusion lesions [117]. As part of the autophagy processes, mitophagy improves the efficiency of the energy production of the cell and ROS scavenging and, in some circumstances, induces apoptosis. MT acted either through the SIRT3-FOXO3 (forkhead box O3)-PRKN signaling cascade in a murine model of atherosclerosis [118] or through the mitochondrial dynamin-like GTPase (OPA1)/AMPK axis in an ischemia-reperfusion model [119]. The overall reduction in ROS suppressed the NLRP3 inflammasome activation in atherosclerotic lesions and the progression of the disease [118]. The vascular smooth muscle cells (VSMC) proliferation is another pathological mechanism in atherosclerosis. In rat aortic smooth muscle cells, MT arrested VCMS at the G1 cell cycle phase, blocking proliferation. It has also been shown that MT upregulates Sestrin2 that regulates cell growth but also diminishes apoptosis. Sestrin2 suppressed the mTORC1 activity in VSMCs, which, in turn, reduced the cleaved caspase 3 level [120]. These apparently contradictory results should be interpreted in the interrelation between autophagy and apoptosis and, up to a certain point, a shared pathway [121]. Autophagy is a survival mechanism initiated by cellular stress, such as excessive ROS, a nutritional deficit, inflammation, etc. The autophagy process enables the cell to reduce metabolism and to initiate necessary repairments. If this is not possible, the apoptosis machinery eliminates the no longer viable cell and maintains tissue homeostasis.

Angiotensin II (Ang II), another marker of high blood pressure, causes oxidant damage in the vascular system by inducing oxidant species generation via the activation of NADPH oxidase. Ang II also stimulates the synthesis of tryptophan hydroxylase, the rate-limiting enzyme of melatonin synthesis [122]. In transgenic mice with overreactive renin-angiotensin-system (RAS), there was an inverted blood pressure (BP) circadian rhythm [123]. The night-time MT secretion correlated negatively with urinary angiotensinogen excretion. All the above plead for a relation between the RAS and MT. Indeed, a treatment with MT of nephrectomised rats ameliorated the RAS activation effects in the remnant kidney. The decrease in blood pressure levels and in interstitial fibrosis markers were associated with a decrease in oxidative stress (evaluated by 8-hydroxy-20-deoxyguanosine) and a higher activity of SOD [124]. The relation was confirmed in a clinical study, in which impaired night-time melatonin secretion was associated with night-time urinary angiotensinogen and albumin [125].

In conclusion, it seems that the antioxidative capacity of MT does play a role in the prevention and/or treatment of the vascular effects of MetS, in conjunction with the other roles of MT in vascular physiology. The contradictory experimental results might depend on the distribution of MT receptors in the peripheral vessels (from adventitia of the large vessels to the endothelium), on the antagonistic effects of MT1 (vasoconstriction) and MT2 (vasodilation) [121,126] and on the level of MT used in different experiments. For example, only micromolar, but not picomolar quantities of MT were able to inhibit the vasodilatation induced by lipopolysaccharides [127]. Last, but not least, the contribution of the other effects of MT beyond the antioxidative one, which are so much influenced by the cellular milieu, cannot be excluded. The complex effect of MT on the blood vessels was illustrated, in healthy subjects, by the different effects of MT on the blood flow, according to the vascular area [126]. After the acute ingestion of MT, there was a reduction in the renal flow, in contrast with an increase in the forearm area and with no effect on brain circulation.

In what concerns the chronodisruption and low MT in night shift workers, a clinical study showed a negative correlation between the diurnal levels of melatonin and the velocity of the pulse wave, as an expression of the vascular distensibility [128]. An indirect proof of the beneficial effect of MT secretion during the night time is the result of a meta-analysis of the randomized controlled studies on the effect of MT on nocturnal blood pressure [129]. The controlled release of MT, a formulation which mimics the physiological pattern of the endogenous hormone secreted during the night, was the only supplement with a significant reduction in both the systolic and diastolic nocturnal blood pressure.

### 3.4. Antioxidative Effects Mediated by MT in Insulin Resistance and Diabetes

Studies have suggested that a desynchrony in the circadian rhythm could trigger metabolic abnormalities. The acute effects of desynchrony are impairment in some physiological processes (e.g., insulin sensitivity, cortisol and melatonin secretion, blood pressure and cardiac modulation by the autonomous nervous system) [129,130,131,132]. In over 40 h of extended wakefulness, polar metabolites had a rhythmic or linear variation. Significant tendencies were identified at both the individual and group level, but a high inter-individual variability was noticed [133], suggesting that other factors are also involved.

The MT receptor gene polymorphisms are related to insulin resistance and diabetes genetic risk in Asian [134], Caucasian [135] or Native Americans [136]. For example, the GG polymorphism of the MTNR1B gene was associated with higher stimulated glucose after a 3 h oral glucose test due to the lower insulin and peptide C early phase secretion in both diabetic and non-diabetic subjects [137]. A meta-analysis of 21 studies showed that the MTNR1B rs10830963 variant increases the risk of type 2 diabetes mellitus [138].

The randomized controlled studies which showed the efficiency of MT treatment in reversing components of MetS in polycystic ovary syndrome [139] or in reducing the metabolic side-effects of antipsychotic drugs [140,141,142] are also strong arguments for a relation between metabolic disorders and MT.

Meanwhile, in type 2 diabetes (T2D), the MT, total antioxidant capacity and SOD levels are lower [143]. In experimental models of T2D, a reduction in MT was noticed mainly during the night time. [144], which makes the relation with night shifts even more relevant. Hyperglycemia promotes an excessive ROS generation in various ways: glucose autooxidation [145], activation of the Nf-KB and the pro-oxidative molecules, epigenetic modifications targeting the expression of SOD [146] or of the Nrf2 gene [147], are relevant examples. In front of oxidative aggression, the β-cells have a weak antioxidant capacity. Compared to the liver, the antioxidant gene expression in pancreatic β-cells is almost half [148]; therefore, molecules with a proven anti-oxidant effect should be of high interest.

Several studies have showed a decrease in oxidative stress in diabetes after a MT treatment in parallel with an increase in insulin sensitivity and/or a decrease in insulin resistance. In one study, after 8 weeks of combined treatment (i.e., MT and magnesium), the total antioxidant capacity was significantly increased [139]. In another randomized controlled study, after 12 weeks of MT treatment, significant increases in glutathione and decreases in MDA were recorded [149]. During the animal testing of new agonists of MT, the insulin sensitivity, increase in MDA and decrease in SOD and glutathione-peroxidase observed in high-fat-sucrose-diet rats were reversed [150].

Oxidative stress reduction and recovery of the mitochondrial function after a MT treatment was also observed in experimental research. For example, in a podocyte injury model developed by similarity with the diabetic nephropathy lesions, the intracellular ROS reduction and the restoration of the mitochondrial membrane potential had an anti-apoptotic effect on the cell damage induced by angiotensin II [151]. In fructose-induced MetS in hamsters, MT restored the triglycerides, total cholesterol and LDL-cholesterol to the initial levels [152]. The effect of MT was also manifest in significantly reducing the lipid peroxidation and restoration of the total antioxidant capacity in the Harderian gland. The reduction in oxidative stress was sufficient enough to inhibit autophagy.

T2DM is the result of insulin resistance, defective insulin secretion and/or a high rate of pancreatic β-cell apoptosis. In experimental T2D, an increase in insulin is associated with a decrease in MT [153]. This action is mediated by both MT1 and MT2. There are different cellular pathways activated by MT, including inhibitory signals (via adenylatcyclase/cadenosine monophoshate and guanylatcyclase/cyclic guanosine monophosphate signaling), but also stimulatory signals (mediated by inositol-triphosphate). At least in animal models, the inhibitory effect is predominant [154]. MT prevents apoptosis of the pancreatic β-cells through enhanced endogenous antioxidant defense [155]. In this experiment, pancreatic β-cells were maintained in glucotoxic and glucolipotoxic conditions. MT normalized the increased levels in Mn-SOD and catalase in the glucotoxic conditions, increased these antioxidants in the glucolipotoxic ones and restored, in both circumstances, the pancreatic secretion of insulin [155]. The same protector effect was noticed in experiments using the proteotoxicity-induced apoptosis of β-cells [156]. These experimental data substantiate the results of a recent meta-analysis, in which MT supplementation improved glycemic control in T2D [157].

## 4. Conclusions

There is extensive research showing that chronodisruption affects MetS. Oxidative stress is a common mechanism for the disorders generated by MetS and MT is a promising molecule to prevent or contribute to the comprehensive approach of these patients. Further research should define how endogenous and exogenous MT should complement each other in order to assure the maximum efficacy in MetS related to working in shifts.

## Data Availability

:Not applicable.

## Supplementary Materials

The following supporting information can be downloaded at: https://www.mdpi.com/article/10.3390/antiox12040959/s1. Table S1: The relation between shift work and MetS – epidemiological studies [158,159,160,161,162,163,164,165,166,167,168].
