# Supplementary material for: Impaired Melatonin Secretion, Oxidative Stress and Metabolic Syndrome in Night Shift Work
Source: Antioxidants (Basel). 2023 Apr 19;12(4):959. doi: 10.3390/antiox12040959 (PMC10135726; doi:10.3390/antiox12040959)
Supplement: Supplementary file 1 [file antioxidants-12-00959-s001.zip › antioxidants-2312373-SI.pdf]

Table S1. The relation between shift work and MetS – epidemiological studies

Components of metabolic syndrome	Study Design	Population	Main results	Confounders
One component				
Large waist circumference [1]	Longitudinal, (5 years follow up)	934 healthy Korean female nurses, 20–39-year-old, 33.8% shift workers	In the 20–29-year-old group, shift work-associated obesity: OR= 3.21, CI 1.29–7.98.<br>In the 30–39-year-old group the multivariate analysis showed a statistically non-significant relation.	Work schedule, follow up period, exercise
High blood pressure [2]	Longitudinal (14 years of follow-up)	5338 Japanese male workers in a steel company, (2748- shift workers, 3963-day workers)	Shift work increases the systolic blood pressure as following: by ≥10%, OR=1.15, CI 1.07-1.23; by ≥15%, OR=1.21, CI 1.12 -1.31); by ≥20%, OR=1.15. CI 1.04 -1.28); ≥25%, 1.20, CI 1.06-1.37); and by ≥30%, OR=1.23, CI 1.03- 1.47.<br><br>Shift work increases the diastolic blood pressure were: by ≥10%, OR=1.19, CI 1.11- -1.28; by ≥15%, OR=1.22, CI 1.13-1.33); by ≥20%, OR=1.24, CI 1.13 -1.37; and by ≥25%, OR= 1.16, CI 1.03 -1.30	Age, body mass index, haemoglobin A1c, total serum cholesterol, creatinine, aspartate aminotransferase, γ-glutamyl transpeptidase, uric acid, drinking habit, smoking habit, and habitual exercise
High blood pressure [3]	Longitudinal (mean follow-up 12.7 years)	10,173 Japanese male from a manufacture (9209 daytime workers, 964 three shift workers)	Shift work is a significant risk factor for hypertension: HR: 1.97, CI 1.79-2.18	Age, smoking, drinking, physical activity, systolic and diastolic blood pressure at baseline, BMI at baseline and increase in BMI during follow up
High blood pressure [4]	Longitudinal, (10 years of follow up)	2151 workers from an aluminium plant	Elevated hazard ratios (HR) observed for all levels of recent NS work compared with non-night workers, and among all levels of rotational work compared with those working <1% rotational work.<br><br>Mostly NS work and frequent rotations had the highest HR for high blood pressure = 3.99, CI 1.64-9.68	Job grade, race, annual health status, calendar year, exposure prior to the previous year, BMI and smoking status
Hyperglycaemia (T2D) [5]	Longitudinal (8 years follow-up)	28,041 from Black Women's Health Study (BWHS) –general population; NS workers compared to never having worked the NS	Duration of NS work increased the incidence of T2D as following:<br>• 1–2 years: HR =1.17, CI 1.04-1.31<br>• 3– 9 years, HR= 1.23, CI 1.06-1.41<br>• ≥10 years HR= 1.42, CI 1.19-1.70 (p-trend <0.0001)	Age, time period, family history of diabetes, education and neighbourhood socioeconomic status.
Hyperglycaemia (T2D) [6]	Longitudinal (15 years follow-up)	28,731 workers from Danish Nurse Cohort (including retired and working nurses)	Higher risk of T2D compared to nurses who worked only day shifts, as following:<br>• nurses who worked NS: HR =1.58, CI 1.25-1.99<br>• nurses who worked evening shifts: HR= 1.29, CI 1.04- 1.59<br><br>Nurses working rotating shifts had a non-significant increase in T2D risk.	Age, smoking status, smoking intensity, physical activity, alcohol consumption (g/week), intake of fatty meat, marital status, employment status, acute myocardial infarction, hypertension, fruit and vegetables intake and BMI

**Two components**				
**Large waist circumference and high blood pressure [7]**	**Cross-sectional**	**1838 women working in a semiconductor manufacturing factory in North Taiwan; (working on fixed 12 h night shifts compared to day shift**	Women working on fixed 12 h night shifts had significantly elevated odds ratios for:<br>• **obesity: OR= 2.7, CI 1.6–4.5**<br>• **central obesity: OR= 2.9, CI 1.7–5.1**<br>• **high blood pressure: OR= 2.3, CI 1.2–4.4**	**Age, smoking, drinking, education, and duration of work**
**Large waist circumference, high blood pressure, MetS [8]**	**Longitudinal (Average follow-up period 2.90 ± 1.63 years)**	**5775 healthy Taiwanese hospital workers (day workers n=3666, night shift workers n=2109)**	**High blood pressure (OR = 1.15, CI 1.01- 1.31) was the only component of MetS associated with increased number of night shifts.**<br><br>**Night shift versus day shift increased the risk for:**<br>• **MetS: OR = 1.40, CI = 1.06–1.85**<br>• **High waist circumference: OR = 1.44, CI 1.17–1.78.**	**Age, sex, occupation, working hours, and baseline metabolic risk factors.**
**Low HDL-C, high triglycerides [9]**	**Cross-sectional**	**665 day workers and 659 three-shift workers in two plants**	**Shift work was associated with:**<br>• **high triglycerides: OR =1.40, CI 1.08–1.83**<br>• **low HDL-C: OR= 2.03, CI 1.18–3.48**	**Age, socio-economic factors, physical activity, current smoking, social support and job strain.**
**Three components**				
**Obesity, high triglycerides, low HDL-cholesterol [10]**	**Cross-sectional**	**27,485 participants from the general population (7909 shift workers, 19576 day workers)**	**Obesity risk in shift workers:**<br>• **women: OR 1.39, CI 1.25-1.55**<br>• **men OR 1.44, CI 1.27-164;**<br><br>High triglycerides risk in shift workers:<br>• **women: OR= 1.13 , CI 1.02-1.25**<br>• **men: OR = 1.12, CI 1.01-1.24.**<br><br>**Low HDL-C risk:**<br>• **women: OR 1.26, CI 1.03-1.53**<br>• **men: OR=1.15, CI 0.96-1.38**	**Age and socioeconomic group.**
**All MetS components**				
**MetS [11]**	**Longitudinal (3 years follow-up)**	1677 Japanese male employees from a car manufacture company (day time workers = 868; 2 shift workers (shifts starting at 06:30 or 15:00 hours) = 686; 3 shifts workers = 99	**MetS risk in 2 shift workers: OR= 1.43, CI 1.05-1.95**<br><br>**There were no significant differences between 3 shifts and daily shift workers.**	Uric acid, Insulin (log), smoking, habitual drinking, habitual exercise
**MetS [12]**	**Cross-sectional**	**26,382 workers general Chinese population (9,088 were shift workers, 17,240 day workers)**	**10 years of shift work increases the risk as following:**<br>• **High TG: OR = 1.03, CI 0.97-1.09**<br>• **Low HDL-C: OR=1.04, CI 0.96-1.12**	**Age, gender, BMI, race, marital status, education, current smoking status, passive smoking, current drinking status, tea or coffee consumption, life**

			• **Hyperglycaemia: OR = 1.05, CI 1.01-1.09**<br>• **Large waist circumference: OR=1.13, CI 1.04=1.22**<br>• **High blood pressure: OR=1.06, CI 1.03-1.09**<br>• **MetS = 1.1, CI 1.01-1.2**	**stress, physical activity, and retirement duration.**
**MetS [13]**	**Longitudinal, average 6.6 years of follow up**	**1529 Belgian male employees from private companies, administration and one bank; shift workers (either 2 or 3 rotating shifts) versus daily workers**	**Increased risk for MetS in rotating shift workers:<br>OR =1.46, CI 1.04–2.07<br><br>Cumulative effect of ≥ 20 years rotating shifts on the development of MetS: OR= 1.82, CI 1.23-2.69 (adjusted only for age)**	**Age, smoking, physical activity outside work, educational level, job strain, physical job demands, and waist circumference, diastolic blood pressure, HDL cholesterol at baseline**
**MetS [14]**	**Longitudinal, prospective 15 years follow up**	**1757 French employees, from different occupational sectors (768 day shift and 989 current or former shift workers).**	MetS associated with shiftwork = OR 1.78, CI 1.03–3.08	**Age, sex, socioeconomic status, smoking, alcohol, stress, and sleep difficulty.**

MetS= metabolic syndrome; T2D= type 2 diabetes; NS= night shift OR= odds ratio; HR= hazard ratio; CI= 95% confidence interval; TG = triglycerides; HDL-C: high density lipoprotein cholesterol

References:

1. Lee, G.-J.; Kim, K.; Kim, S.-Y.; Kim, J.-H.; Suh, C.; Son, B.-C.; Lee, C.-K.; Choi, J. Effects of Shift Work on Abdominal Obesity among 20-39-Year-Old Female Nurses: A 5-Year Retrospective Longitudinal Study. *Ann Occup Environ Med* **2016**, *28*, 69. https://doi.org/10.1186/s40557-016-0148-6.
2. Suwazono, Y.; Dochi, M.; Sakata, K.; Okubo, Y.; Oishi, M.; Tanaka, K.; Kobayashi, E.; Nogawa, K. Shift Work Is a Risk Factor for Increased Blood Pressure in Japanese Men: A 14-Year Historical Cohort Study. *Hypertension* **2008**, *52* (3), 581–586. https://doi.org/10.1161/HYPERTENSIONAHA.108.114553.
3. Kubo, T.; Fujino, Y.; Nakamura, T.; Kunimoto, M.; Tabata, H.; Tsuchiya, T.; Kadowaki, K.; Odoi, H.; Oyama, I.; Matsuda, S. An Industry-Based Cohort Study of the Association between Weight Gain and Hypertension Risk among Rotating Shift Workers. *J Occup Environ Med* **2013**, *55* (9), 1041–1045. https://doi.org/10.1097/JOM.0b013e31829731fd.
4. Ferguson, J. M.; Costello, S.; Neophytou, A. M.; Balmes, J. R.; Bradshaw, P. T.; Cullen, M. R.; Eisen, E. A. Night and Rotational Work Exposure within the Last 12 Months and Risk of Incident Hypertension. *Scand J Work Environ Health* **2019**, *45* (3), 256–266. https://doi.org/10.5271/sjweh.3788.
5. Vimalananda, V. G.; Palmer, J. R.; Gerlovin, H.; Wise, L. A.; Rosenzweig, J. L.; Rosenberg, L.; Ruiz Narváez, E. A. Night-Shift Work and Incident Diabetes among African-American Women. *Diabetologia* **2015**, *58* (4), 699–706. https://doi.org/10.1007/s00125-014-3480-9.
6. Hansen, A. B.; Stayner, L.; Hansen, J.; Andersen, Z. J. Night Shift Work and Incidence of Diabetes in the Danish Nurse Cohort. *Occup Environ Med* **2016**, *73* (4), 262–268. https://doi.org/10.1136/oemed-2015-103342.
7. Chen, J.-D.; Lin, Y.-C.; Hsiao, S.-T. Obesity and High Blood Pressure of 12-Hour Night Shift Female Clean-Room Workers. *Chronobiol Int* **2010**, *27* (2), 334–344. https://doi.org/10.3109/07420520903502242.
8. Cheng, W.-J.; Liu, C.-S.; Hu, K.-C.; Cheng, Y.-F.; Karhula, K.; Härmä, M. Night Shift Work and the Risk of Metabolic Syndrome: Findings from an 8-Year Hospital Cohort. *PLoS One* **2021**, *16* (12), e0261349. https://doi.org/10.1371/journal.pone.0261349.
9. Karlsson, B. H.; Knutsson, A. K.; Lindahl, B. O.; Alfredsson, L. S. Metabolic Disturbances in Male Workers with Rotating Three-Shift Work. Results of the WOLF Study. *Int Arch Occup Environ Health* **2003**, *76* (6), 424–430. https://doi.org/10.1007/s00420-003-0440-y.
10. Karlsson, B.; Knutsson, A.; Lindahl, B. Is There an Association between Shift Work and Having a Metabolic Syndrome? Results from a Population Based Study of 27,485 People. *Occup Environ Med* **2001**, *58* (11), 747–752. https://doi.org/10.1136/oem.58.11.747.
11. Kawada, T.; Otsuka, T. Effect of Shift Work on the Development of Metabolic Syndrome after 3 Years in Japanese Male Workers. *Arch Environ Occup Health* **2014**, *69* (1), 55–61. https://doi.org/10.1080/19338244.2012.732123.
12. Guo, Y.; Rong, Y.; Huang, X.; Lai, H.; Luo, X.; Zhang, Z.; Liu, Y.; He, M.; Wu, T.; Chen, W. Shift Work and the Relationship with Metabolic Syndrome in Chinese Aged Workers. *PLoS One* **2015**, *10* (3), e0120632. https://doi.org/10.1371/journal.pone.0120632.

13. De Bacquer, D.; Van Risseghem, M.; Clays, E.; Kittel, F.; De Backer, G.; Braeckman, L. Rotating Shift Work and the Metabolic Syndrome: A Prospective Study. *Int J Epidemiol* **2009**, *38* (3), 848–854. https://doi.org/10.1093/ije/dyn360.
14. Tucker, P.; Marquié, J.-C.; Folkard, S.; Ansiau, D.; Esquirol, Y. Shiftwork and Metabolic Dysfunction. *Chronobiology International* **2012**, *29* (5), 549–555. https://doi.org/10.3109/07420528.2012.675259.
